# Observations on Aneurism, Particularly with Reference to the Application of a Ligature beyond the Tumor; Being an Abstract of a Clinical Lecture Lately Delivered

**Published:** 1827-06

**Authors:** 

**Affiliations:** *the* Middlesex Hospital.


*Observations on Aneurism, particularly with Reference to the Application of a Ligature beyond the Tumor; being an Abstract of a Clinical Lecture lately delivered by* Mr. Shaw, *at the* Middlesex Hospital.

It would appear, from numerous facts, that, in the greater number of cases, it is only necessary to impede the current of blood through an aneurismal tumor, in order to produce coagulation. But even tying the main trunk leading to the aneurism is not always successful in effecting this, although an apparently slight obstruction to the direct current has been sometimes sufficient.

This leads to the consideration of a very interesting question,—viz. the possibility of causing the blood to coagulate in the sac by tying the artery beyond the tumor; a question which some of you may recollect we also discussed in a lecture last January. Before the operation on the external iliac artery was proposed by Mr. Abernethy, aneurism at the groin was considered, both in this country and on the continent, as a fatal disease, unless it could be cured by pressure. It is true that Brasdor, professor of surgery in the old School of Medicine in France, proposed to tie the artery below the tumor. Desault also recommended this operation; and Deschamps tied the femoral artery in the middle of the thigh for an aneurism at the groin, but the patient died. Although the operation was then given up in France, we have only to read Deschamps' own account of the operation to be satisfied that it should not be taken as a precedent.

Many years after this, Sir A. Cooper tied the femoral artery between the epigastric and the profunda, for an aneurism of the external iliac. The aneurismal tumor diminished in size, the ligatures came away, and every thing promised well. The patient went to the country to recruit his health, but he died suddenly, and it would appear from the aneurism bursting; for, although the body was not opened, a quantity of blood was observed in the cellular membrane of the parts about the pelvis.

It is not improbable that the aneurism in this case might have subsided, had it not been very large before the operation. On considering the probable effect of so directly obstructing the great current of blood through the main artery, we must admit that this operation was founded on just principles, and not to be compared with that performed by Deschamps. But the general opinion was against the operation; and this was expressed, about that time, in very strong language, by the late Allan Burns, of Glasgow. He says, "that there is no point in the treatment of aneurism which ought to be more decidedly reprobated than this: it is absurd in theory, and experience proves that it is ruinous in practice."

This was in 1811; but, notwithstanding the failure of these operations, Mr. Bell used, in his lectures at that time, to insist on the operation being good in principle, and to argue that the facts of anatomy were sufficient to show the cause of these operations having failed. Some of you may remember that, while I was a teacher in Windmill-street, in demonstrating the arteries, and particularly the inosculations of the vessels about the shoulder, I always dwelt on this question. Indeed, the following extract will satisfy you that I have been long interested in it. It is part of a criticism on some continental works on Aneurism, and is inserted in the first Number of the Quarterly Journal of Foreign Medicine, published in 1818. After describing the well-known operation of Deschamps, I remarked, "It will at once be allowed that the question of tying the artery below the tumor cannot be fairly judged of by this case; for an operation so performed, although above the tumor, would have proved fatal. The French surgeons are unanimous in objecting to this operation, (tying the artery below the tumor:) they say that the tumor is rapidly enlarged by it; but a case has occurred in this country where, after a similar operation, the tumor rather diminished." (Sir A. Cooper's case.)

"The question is not now interesting in considering aneurisms at the groin; but it is particularly so in aneurisms of the subclavian artery. The attempt to cure aneurism of the axillary artery by tying the subclavian, has been frequently made both in England and France. Some time ago the operation was successfully performed by Dr. Post, of New York, a gentleman of great talent, and whom we recollect having seen in this country." (Since the paper from which I quote was written, several cases have been successful.) "But how often has this operation failed,—and not so much in consequence of tying the artery, (I here should have said an artery near the heart,) as from the circumstance of there being so many large branches going off from the point at which we must tie it. One cause of our great success in the operations upon the carotid and external iliac arteries, is indubitably the fact of there being no branches going off near to the part where the artery is generally tied. In axillary aneurism, we may tie the artery below the tumor; and, if this be unsuccessful, we may either amputate the arm at the shoulder or tie the subclavian above the tumor; for we have no dread of the ligature of the artery below the aneurism producing the rapid enlargement which the French ascribe to it. Their whole data are taken from Deschamps' operation. We had lately under our care a patient with axillary aneurism, upon whom it was proposed to perform this operation; but the patient being swayed by the opinion of another surgeon, procrastinated so long that the tumor burst, and he died immediately."

This case occurred when I was a very young surgeon. I sent the patient to Mr. Bell: he proposed to tie the artery below the tumor; but the patient, after consulting with Mr. Lynn, would not submit to it.

You may readily imagine that a question of such importance as this, and on which I had written in 1818, has not been forgotten by me: I have indeed taken much interest in the cases in which the operation of tying the artery beyond the tumor has been performed. It is said that there have lately been four instances of this kind, and that three of them have been fatal; while, in regard to the fourth case, (the only one that has survived,) there is reason to suspect that the disease was not aneurism. Were we to rest satisfied with this statement, we should at once conclude that the principle of the operation was bad, and that it ought consequently to be abandoned. But, so far as we have been able to learn, we would rather say that none of the three fatal cases in any way disturbs the question of the principle on which the success of the operation depends.

I presume that the statements made in relation to these cases are correct: if not, it is probable that I shall be set right, as I propose to publish them in the work on Aneurism in which I am now engaged. As I intend to take that opportunity of going minutely into an examination of the first case, (in the Medico-Chirurgical Transactions, July 1825,) I shall at present only observe that, if the delineation of it given by the surgeon who operated be accurate, it is of itself almost sufficient to show that the disease was not aneurism. I shall then also undertake to prove that aneurism of the lower part of the carotid is a very rare disease, and that, when it has taken place, it neither resembles the drawing in appearance, nor in any of its symptoms corresponds with the history of the case alluded to. If I succeed in showing this, then the carotid having been tied in this instance will weigh neither in favour of nor against the proposed operation of tying the artery beyond the tumor.

In the second operation to which I have alluded, the femoral artery was tied in a case which appears to have been similar to that of Deschamps, and analogous to those cases for which the ligature on the external iliac artery has been so successful: I therefore assume, with respect to this likewise, that it was not a case by which we are to judge of the merits of the operation.

The next case is a very extraordinary one. A pulsating tumor in the lower part of the neck was supposed to be an aneurism of the root of the carotid artery. On the 10th of December, an operation, with a view to tie the common carotid, was performed. Eleven days after this, the public were told that the operation had been so completely successful that the patient would soon be able to go home; and, on the 24th of March, a surgeon, whom the operator had assisted on the 1st of March to perform a similar operation, says, in relation to that gentleman's cases, "I may simply remark, that they were cases which, in pursuance of prevailing opinions, would have been left to inevitable death: Mr. Wardrop has therefore the gratification of having saved the lives of two fellow creatures, as well as of having established a principle in the treatment of aneurism, the future benefits of which are almost incalculable." At this time certainly no man could have more apparent reason to exult in what he had done; for he believed that he had discovered the cause of a fatal disorder, and averted its effects by an operation: and, but for the extraordinary circumstances subsequently brought to light, every surgeon would have had cause to exult. The congratulation, however, was premature; for within a day or two of the date of the last so favourable report, the patient died; and, in the course of a short time afterwards, the other patient, (operated on March the 1st,) of whom a most favourable report is given on the 24th of March, also died.

Although it was reported that the tumor had subsided, and that the distressing symptoms were relieved by cutting down on the carotid, the fatal issue of both cases in so short a time might lead to the supposition that the principle of tying the artery beyond the tumor was radically bad: but, happily for the credit of the operation, the bodies were examined after death. In the first case, no aneurism of the carotid, nor of any of the great vessels, was found, but the heart was in that condition which is called hypertrophy. This is nearly the substance of the account published, and the statement was corroborated by a gentleman who had an opportunity of examining the parts carefully, and whom you all knew as a most intelligent and diligent student. But, what is still more extraordinary, the carotid, which had been supposed to be tied, was found entire, and without any marks of a ligature having ever been applied to it. The blood, to the day of the patient's death, appears to have passed as freely through it as through any other vessel. As far, therefore, as regards the question at issue, it is clear that this case can have no effect on it. We cannot, however, avoid expressing our surprise that a pulsating tumor in the neck, caused by irregularity in the action of the heart, should, after all that has been said and written on that subject, be mistaken for aneurism. I shall not attempt to account for the phenomenon of the carotid artery being found entire, nor for the good effects which are reported to have been produced by the supposed obstruction of the circulation through it.

In regard to the latter case, of which a most favourable report was given on the 24th of March, I have only heard that the patient died of hemorrhage since the date of that report.

I shall now recall to your recollection the case of Gordon the blacksmith, who lay in Clayton's ward. You may remember that, when this man came into the hospital, he had a pulsating tumor, nearly the size of a hen's egg, extending from under the sternal portion of the clavicle to the edge of the trapezius, and rising towards the thyroid cartilage. Before he came into the house, he had been taking mercury under the care of a person in the neighbourhood, his disease being supposed to be rheumatism; and, although it was said he had only taken a few pills, he was in such a state of salivation that, had an operation been proper, it could not have been performed at that time. However, there was little difficulty of ascertaining that the disease was not aneurism of the carotid, and there was every reason to believe that the innominata was affected. I will not at present enter into a discussion of the grounds on which I formed this opinion: I may, however, remark that the character of the pulsation in the right carotid and right humeral arteries exactly corresponding, and of its being different from that in the vessels of the left side, were the principal facts which led me to infer that the common trunk was affected. From the first, I determined that all we could do was to treat this patient on the depletory system. Some of you know that the unwarrantable conduct of strangers, who, although medical men, visited the patient clandestinely, made it difficult for me to pursue this plan of treatment; and, had not the attention which compassion for his situation led me to pay him, induced the poor patient to place some confidence in me, he would have probably yielded to the attempts which were repeatedly made to carry him to another surgeon, for the purpose of having the carotid artery tied. Having been informed of these circumstances, I told the patient, at one of our public visits, that an operation in his case was not to be thought of. You may remember his reply: that he would trust to me, and have nothing to do with the gentleman (whose name he mentioned) to whom he had been urged to go.

It is but justice to myself and other hospital surgeons to state this, that you may know to what we are in the present day subjected; for not only did these persons endeavour to destroy my patient's confidence in me, but, failing in that, they have since attempted to injure me in public estimation by a false statement of the case.

The tumor and the symptoms of dyspnœa increased; the pulsation in the carotid gradually diminished, and at length ceased altogether; but, notwithstanding this, the tumor continued to enlarge. The breathing became more difficult; and in the beginning of December it was evident the man was dying. His wife had an unfortunate horror at the idea of her husband dying in an hospital, and insisted on carrying him away, although the danger of his expiring on the road was pointed out to her. The poor man begged I would visit him at home. I saw him a few hours afterwards. His breathing was now very laborious; and, on calling next day, I found that he had expired suddenly: the people who were sitting by him having heard him groan, observed the tumor gradually sink and become flattened.

I regret that it is not in my power to exhibit to you the condition of the vessels; I have only been able to learn that the aneurismal tumor was, as I supposed, in the innominata, and that the right carotid had been obliterated.

But it is incumbent on me to satisfy you that it was not from indolence nor from carelessness on my part, that I am unable to prove this to you by an inspection of the aneurism. On first applying for leave to examine the body, his wife was not at home; and I therefore went again, to endeavour to obtain her consent. To my astonishment, I was told that it had been already examined by some young men, who had said to the friends that "it would be the same as if I were present." Hoping that the parts might not have been removed, I requested to see the body. On opening the incision, I found that the subclavian and carotid arteries had been removed; and now an old nurse whispered to me that she had allowed them to take *the substance* to my house, for me to see it. I confess I had little expectation that men who acted so unwarrantably in using my name would send the parts to me; and accordingly, in the course of the day, I discovered that the preparation had been immediately taken to another surgeon. I shall not mention the young man's name who was the principal agent in this most unprofessional transaction: I am happy to say he never was a pupil here.

I wrote to request that my colleagues and you might have an opportunity of seeing the state of the parts in a case in which we had been so much interested; but to this day I have not seen it, although several of my friends had an opportunity of doing so. This preparation has been exhibited before many persons as an example of an aneurism which might have been cured by operation.

Although it is impossible to look upon this transaction without some feelings of indignation, yet I trust that I shall not allow these to bias me in the discussion of this important question. Fortunately the opinions of those who think that an operation would have been admissible are placed on record; and as the only former occasion on which I thought it necessary to notice the systematic slander with which I have been assailed related to an important question with regard to hemorrhage after lithotomy, so now I enter on this discussion only because it may be converted to your instruction. The passages run as follows:—"Nothing further was done, excepting to order a chalk mixture or two for a purging which had supervened; and, being determined to remain no longer under this treatment, the patient left the hospital on the 4th December, intending to go to Panton-square: but it was too late, the unfortunate man survived only twenty-four hours after leaving the Middlesex Hospital, having been there about six weeks." "A more instructive dissection could scarcely be seen: it shows how much might have been done by an energetic surgeon, and how much might be sacrificed to timidity or something worse." "The success which attended Mr. Wardrop's operation of tying the carotid above the aneurism ought to have induced the illuminati of the Middlesex to have attempted a similar mode of treatment: they might first have tied the carotid, and, if that were found insufficient to have stayed the progress of the tumor, they should have afterwards tied the subclavian, rather than have given the man up to his fate. It will be seen, indeed, from the report, that, ten days previous to the patient's leaving the hospital, the carotid artery had become obliterated; and that fact was not unknown to Messrs. Bell and Shaw. The preparation is at present in the possession of Mr. Wardrop."

If tying the carotid or the subclavian in such cases is to be considered the proof of being an "energetic surgeon," I trust I shall never merit such a character. The mere operation of tying a large artery can appear a great and bold achievement only to those who are conscious of being deficient in anatomical knowledge, or *who have had the misfortune to attempt the operation without succeeding in its accomplishment.* The true test of skill consists in judging correctly of the time and circumstances under which such an operation ought to be performed.*

To say that both the carotid and subclavian should have been tied in the case alluded to, is so absurd, that, to combat this idea, it is only necessary to attend to the anatomy of the vessels. With regard to the carotid, indeed, the preparation which was taken from me—I had almost used a stronger expression,—shows that, although that vessel had been obliterated by the pressure of the tumor, and the current of blood consequently no longer passed through it, still this had produced no salutary effect on the tumor, for it rapidly increased, and soon afterwards burst. Nor is the case treated by me a solitary one. Sir Everard Home has mentioned an instance in which the carotid became obliterated from the same cause; and there are two magnificent specimens in the Museum of the College, which I advise you to examine, as they are sufficient to prove that tying the carotid artery can have little or no good effect on an aneurism of the innominata.

I place before you a drawing from Scarpa, of an aneurism in the lower part of the trunk of the carotid; but this you see, instead of having formed an obscure tumor at the lower part of the neck, mounts as high as the angle of the jaw. I shall take another opportunity of proving that, although there is frequently a dilated state of the origin of the carotid in elderly persons, and which is not dangerous as a local affection, but only symptomatic of general disease in the arterial system, true aneurism of the carotid is a very rare disease, excepting at its separation from the subclavian, (being then in fact aneurism of the innominata,) or at its bifurcation. The connexions of the deep fasciæ will likewise show that, if aneurism should take place in the root of the carotid, the tumor must ascend towards the jaw, and form an external tumor, quite different in appearance from the drawing given in illustration of the case said to have been carotid

* It has, I know, been said that I was *afraid* to perform the operation. This is true; but I was afraid only because I knew that the operation would be injurious. It is a most dangerous doctrine that, if the surgeon thinks his patient must die of the disease, he is therefore justified in performing any wild experiment, with however little prospect of success. At present there are some individuals among us who are endeavouring to acquire notoriety by practising operations the most desperate, and I hesitate not to say the most unwarrantable.

aneurism, and alleged to have been cured by tying the artery beyond the tumor.* Indeed, from these and other considerations, I am led to believe that that case was not one of aneurism at all.

The operation of tying the artery beyond the tumor can be seldom available or proper even in cases of true carotid aneurism; yet, notwithstanding the late unsuccessful results, it should not be lost sight of in cases more judiciously selected, and under circumstances more favourable to its performance.

---

After lecture, one of the gentlemen present showed Mr. Shaw a plan which had been made of the arteries, on the dissection of the patient who had been operated on on the 1st of March, (Mr. Lambert's case.) He remarked that at first view it appeared somewhat similar to an aneurism; but, after finding that the tumor was described in the published statement to be only half an inch in breadth, he said this showed that it had only been such a dilatation as was frequently observed in elderly persons. He remarked that the circumstance of the channel through the artery having been obstructed, although it had been tied with "fisherman's silk," as in the other fatal case where the vessel remained pervious, proved the correctness of the general opinion, that not the carotid, but something else, had been tied in that instance. There was, he remarked, a curious anomaly in the history of these two cases. In both a pulsating tumor was said to have existed at the lower part of the neck; and in both the symptoms supposed to have been produced by these tumors were described as having been completely relieved by an operation: but, on dissection, a dilatation of the carotid, very different from true aneurism, was found in the one; while the other proved to be a case of disease of the heart, without any aneurism, and with but very inconsiderable dilatation. In the former the carotid was found to have been tied, and the consequent obstruction to the course of the blood through the vessel was stated to have had the same effect in mitigating the sufferings of this patient, as the mere cutting down to look for it had in relieving the symptoms produced by enlargement of the heart in the other!

* Med.-Chir. Transactions, vol. xiii.

